# Biomedical moral enhancement for psychopaths

**DOI:** 10.1111/bioe.13373

**Published:** 2024-11-04

**Authors:** Junsik Yoon

**Affiliations:** ^1^ Department of Ethics Education, College of Education Seoul National University Seoul Republic of Korea

**Keywords:** biomedical moral enhancement, informed consent, medical benefit, neuroethics, psychopathy, right to integrity, stigma

## Abstract

This study examines the ethical permissibility of biomedical moral enhancement (BME) for psychopaths, considering both coercive and voluntary approaches. To do so, I will first briefly explain what psychopaths are and some normative implications of these facts. I will then ethically examine three scenarios of BME for psychopaths: (1) coercive BME for non‐criminal psychopaths, (2) coercive BME for psychopathic offenders, and (3) voluntary BME for psychopathic offenders. I will argue that coercive BME for non‐criminal psychopaths is ethically problematic due to issues of cost, invasion of privacy, and stigmatic effects of compulsory diagnosis. Similarly, I will argue that coercive BME for criminals is impermissible due to violations of the rights to bodily and mental integrity. However, I will show that voluntary BME for offenders may be ethically permissible under certain conditions, challenging the critique that the consent of vulnerable prisoners cannot be considered fully voluntary. I argue that when an offender is provided with sufficient medical and legal information, incentives such as the possibility of parole review based on BME results do not preclude the voluntariness of consent. Ultimately, I aim to advance the debate on BME for psychopaths by delineating and defending conditions for the ethical permissibility of voluntary BME.

## 1 INTRODUCTION

Biomedical moral enhancement (BME) can be defined as a partial improvement of morality‐related functions through biomedical interventions.^1^ Some argue that if safe and effective BME technologies are developed, their use should be mandatory.^2^ This strong position has been widely criticized, especially with respect to issues of freedom and autonomy.^3^ Voluntary BME is also often criticized for its inherent problems of instrumentalism and self‐paternalism.^4^ However, if the intervention is not a direct way of inducing or increasing the frequency of specific behaviors, voluntary BME seems to have room to be ethically permissible.^5^

Psychopaths are the individuals in whom the positive effects of BME can be most dramatically confirmed. Many empirical studies attribute their antisocial characteristics to abnormalities in morality‐related functions, such as emotion regulation or empathy. Given the significant harm that psychopaths inflict on society, the potential benefits of improving their moral competence through BME are also substantial. In this context, some argue that BME can be forced on psychopaths.^6^ For those who exhibit psychopathic tendencies, the notion of coercing BME seems particularly compelling, as they are already subjected to the imposition of punishment.

In contrast, there is also the argument against voluntary BME for psychopathic offenders.^7^ This position highlights a crucial issue in bioethics: whether the nominal consent of vulnerable individuals, such as prisoners, can be considered fully voluntary. If a psychopathic prisoner were to consent to BME, it would probably be motivated by external reasons, such as parole, rather than by a genuine desire for moral improvement. Consequently, there is no genuine voluntariness or respect for autonomy; rather, there is manipulation through incentives. Even the notion of voluntary BME appears to be challenged in the case of psychopathic offenders.

The aim of this study is to defend the position that while coercive BME is ethically impermissible, voluntary BME can be ethically permissible in the context of psychopaths. To this end, in Section 2, I will first examine the fact that psychopaths have serious dysfunctions in emotional functions related to morality based on previous studies and then explore the normative implications of this. In Sections 3 and 4, I will argue that coercive BME cannot be justified, not only in the case of non‐criminal psychopaths (Scenario 1) but also in the case of psychopathic offenders (Scenario 2). In Section 5, I will show how psychopathic prisoners can voluntarily consent to BME despite their vulnerability.^8^

## 2 NORMATIVE IMPLICATIONS OF THE CONCEPT OF PSYCHOPATHY

The first clinical description of psychopathy was reportedly made by the French psychiatrist Philippe Pinel in the early 19th century. He defined the behavioral pattern of psychopathy, which he characterized as “utter remorselessness and a complete lack of restraint,” as “insanity without delirium.”^9^ Hervey Cleckley's 1941 publication, *The Mask of Sanity*, presents a more contemporary conceptualization of psychopathy, including its immoral characteristics. Simon Baron–Cohen offers a concise summary of Cleckley's characterizations of psychopathy: “superficial charm, a lack of anxiety or guilt, undependability and dishonesty, an inability to form lasting intimate relationships, a failure to learn from punishment, poverty of emotions, a lack of insight to the impact of their behaviour, and a failure to plan ahead.”^10^ Since Pinel and Cleckley, numerous studies have been conducted to understand the causes of psychopathy and to diagnose psychopaths from non‐psychopaths. The most widely used tool for diagnosing psychopathy is the PCL‐R (Psychopathy Checklist‐Revised) developed by Canadian psychiatrist Robert Hare in 1991. It consists of 20 items, and Hare diagnoses individuals as psychopaths when the total score of each item is 30 or more.

Many researchers have focused their attention on the role of affective factors. Some have made a clear distinction between the concept of antisocial personality disorder in the Diagnostic and Statistical Manual of Mental Disorders (DSM) and the concept of psychopathy. Both have antisociality in common. But while the former uses only outwardly objective symptoms as diagnostic criteria,^11^ the latter includes a disorder in a specific function, namely emotional processing due to brain abnormalities.^12^ This causes the inability of the violence inhibition mechanisms. In this regard, Baron‐Cohen states that there is abundant evidence of “abnormalities in the empathy circuits of the brain” of psychopaths. Based on this, he argues that “instead of using the term ‘evil’ we should talk about reduced (or even absent) empathy.”^13^ Psychopathy may be a case of Moral Deficiency Disorder (MDD), a disorder “characterized by a deficit of empathy,”^14^ as proposed by Sarah Carter. Carter suggests that in the future, if methods are developed to ease the dysfunction experienced by psychopaths significantly, psychopathy could be formally diagnosed as a disorder or disease, and interventions to mitigate it could be recognized as treatment. Based on these empirical and philosophical researches on psychopathy above, I anticipate the potential medicalization of psychopathy with cautious optimism.

In light of the preceding discussion, two key points emerge from a normative perspective. First, psychopaths are a kind of moral incompetence with serious problems in emotional functions that inhibit unethical and illegal behavior. Second, more importantly, the psychopaths themselves cannot be considered to have caused the fatal brain dysfunctions that are identified as the cause of these symptoms. If these claims are valid, it may be necessary to reconsider our general moral intuitions about psychopaths and the manner in which they are treated within the context of institutional frameworks. In particular, there is room for doubt about the position that the mere existence of psychopaths and their antisocial tendencies and behaviors can be the subject of blame or that the parties concerned can be held responsible for them.

One objection to this perspective is that the responsibility of psychopaths should be held because not all psychopaths commit crimes. This is indeed the case; there are so‐called prosocial and successful psychopaths in our society, as illustrated by the example provided by James Fallon below. However, it is challenging to consider the possibility of becoming prosocial psychopaths as an achievable goal through individual effort. The case of James Fallon, who has gained recognition as a “psychopathic neuroscientist,” is illustrative of this context. Through his own brain scan image, he learned that his brain activation pattern belonged to the psychopathic type, and later confirmed that many of his ancestors and relatives were murderers. Fallon subsequently proposes what he refers to as the “three‐legged stool theory” of psychopathy. The three legs of this theory are as follows: (1) the unusually low functioning of certain brain areas, (2) the high‐risk variants of several genes, and (3) early childhood abuse.^15^ In his case, fortunately, he did not have the third leg. He thinks he is a lucky psychopath because his nurturing family background allowed him to live as a non‐violent or prosocial psychopath.^16^

If it is difficult to attribute psychopathy to individual responsibility, then, a restorative and rehabilitative approach, rather than retributive punishment, may be more appropriate for psychopaths.^17^ Moreover, the development of safe and effective BME to cure psychopathy will further increase the realistic possibility of this institutional transition.^18^ However, whether BME can be forced on psychopaths is a completely different question. While various ethical issues, including physical side effects, should be discussed, to focus on the issue of coercion and voluntariness, we will proceed here with the assumption of safe BME. I argue that even in the case of psychopaths, regardless of whether they have committed crimes or not, coercive BME is impermissible, while voluntary BME is permissible. To demonstrate this, I will examine three scenarios: (1) coercive BME for non‐criminal psychopaths, (2) coercive BME for psychopathic offenders, and (3) voluntary BME for psychopathic offenders.

## 3 SCENARIO 1: COERCIVE BME FOR NON‐CRIMINAL PSYCHOPATHS

The necessary condition for the coercive BME could be a diagnosis of psychopathy. Currently, psychopathy is diagnosed by professionals only in criminals who are highly antisocial. The criterion of crime is practical, in that it reduces the scope of the costly diagnosis. Scenario 1, however, extends the scope to non‐criminals. The problem is that the diagnosis would also have to be coercive. Unlike criminals, mandatory diagnosis for non‐criminals appears problematic. As I will discuss in more detail below, there are legitimate ethical concerns about the high cost, privacy infringement, and (self‐)stigma effects. Therefore, in this section, I will focus on these three problems, posed by the mandatory diagnostic procedure for non‐criminals. The common problem in Scenario 1 and Scenario 2, the issues directly caused by forced BME, will be addressed in the next section.

Based on the three‐legged stool theory of psychopathy, let us imagine a compulsory psychopathy diagnosis. It would first require an examination of the low‐functioning areas of the brain that are most directly responsible for psychopathy. It would also include an investigation of the genes and nurturing environment that may lead to such brain dysfunction. As previously mentioned, the scope of diagnosis would be extended to the entire population. Such a procedure would necessitate a significant investment of human and material resources. Even if it were feasible, it is questionable whether it would be cost‐effective. From a justice perspective, it could be also interpreted as an inappropriate distribution of public funds. Of course, the cost‐benefit ratio may improve with technological advancement. Therefore, a separate review of ethical issues that are difficult to accept is necessary.

One of these is privacy infringement. It is difficult to find a universally accepted definition in academia, but privacy has been discussed, especially as personal information.^19^ Privacy as personal information is important, especially from a normative perspective, because it is deeply connected to the formation and maintenance of personal identity and interpersonal relationships.^20^ When sensitive personal information, such as sexual orientation, family background, disabilities, and medical conditions, is compromised, a person's identity and interpersonal relationships can be severely damaged. Thus, we have ethical reasons to protect their privacy as personal information. The psychopathy diagnosis discussed in this section, which involves the compulsory collection of an individual's physical and mental information, is directly related to the violation of privacy as personal information.

Let us revisit Scenario 1 based on the discussion above. If an individual is diagnosed as a psychopath, few would be inclined to disclose the data that forms the basis of that diagnosis. This information is of significant value and therefore worthy of protection. It is reasonably foreseeable that the disclosure of a psychopathic identification will have a significant negative impact, especially on interpersonal relationships. In Scenario 1, the purpose of diagnosing psychopaths is not merely to isolate and control them, but to improve their dysfunctions through safe and effective BME, thereby reducing the risk of criminal behavior and facilitating their integration into society. Consequently, the government should protect the information on whether someone is a psychopath or not, obtained through the mandatory diagnostic test.

However, can the relevant information be effectively protected? The diagnosis in Scenario 1 is far from a secret test for a limited number of subjects. All citizens are aware that a widespread psychopathy diagnosis is being conducted and will have a strong interest in the diagnostic results of not only themselves but also others. Furthermore, those identified as psychopaths will undergo BME procedures ranging from prescription drug administration to periodic injections and brain surgery. Even if the state does not disclose the information, close individuals such as family and friends are likely to discover the selection results and the subsequent BME. Even if the BME results are highly effective and the individuals' prosociality is significantly increased post‐BME, the impact of the state's psychopath diagnosis on social relationships will be severely detrimental. Despite medical advancements that have made AIDS a manageable chronic condition, social exclusion and discrimination still occur simply because someone identifies as a sexual minority, even in the absence of actual threat factors. The negative consequences of being labeled a psychopath on social relationships will be so severe.

What if it is completely confidential? Even under this unrealistic assumption, the “stigma” must be considered. Generally, the stigma effect refers to the phenomenon in which a person labeled with a negative prejudice or stereotype begins to embody that label.^21^ This effect can occur not only by others but also by oneself, that is, in the case of self‐labeling. The experience of recognizing oneself as a psychopath can lead to such self‐stigma, even if it is done in complete secrecy. Self‐stigma has generally been reported to have adverse effects on psychological well‐being, such as diminished self‐esteem and reduced self‐efficacy. These negative impacts have been observed to persist even after the mental health condition that precipitated the stigma has been ameliorated through treatment.^22^ While empirical investigations specifically examining the ramifications of compulsory psychopathy diagnosis in non‐criminal psychopathic individuals—as postulated in Scenario 1—are sparse, there exists a body of research suggesting that certain factors, including psychopathy and antisocial personality disorder, may predispose individuals to heightened vulnerability in the self‐stigmatization process. This evidence may provide a substantive basis for concern regarding the potential implications of psychopathy diagnosis.^23^

Despite ethical concerns such as invasion of privacy and (self‐)stigmatization, is there a reason for mandatory psychopathy diagnosis? The underlying goal should be crime prevention. However, the standard biological markers of psychopathy used in the diagnoses are merely demographic patterns and are difficult to recognize as a legitimate basis for predicting the future behavior of any given individual. In fact, this problem leads to the philosophical dilemma of the free will debate, but a complex discussion is beyond the scope of this article. Nevertheless, it is worth noting that even Gregg Caruso, who is skeptical of the concepts of free will and the moral responsibility and the basic desert based on it from a deterministic point of view, argues that preemptive intervention, such as specifying and isolating criminals before they commit crimes, is difficult to justify.^24^ This perspective supports that a diagnosis of psychopathy cannot be considered a definitive evidence of future criminal behavior. Furthermore, forced diagnoses have many of the ethical problems discussed so far. Even before forced BME, the forced psychopathy diagnosis required for it alone has problems that are difficult to justify.

## 4 SCENARIO 2: COERCIVE BME FOR PSYCHOPATHIC OFFENDERS

Scenario 2 seems more defensible than scenario 1. Primarily, criminals are already subject to coercive punishment.^25^ Additionally, BME is likely to be more effective than simple imprisonment in achieving important goals, such as recidivism prevention, correction, and rehabilitation. Nevertheless, these facts alone cannot justify coercive BME. For example, consider corporal punishment, such as flogging. This practice is now uncommon due to its violation of the right to bodily integrity (RBI), which is considered the core of the right to liberty guaranteed in most countries by constitutions. Since coercive BME also involves some form of physical intervention, its legitimacy must be examined from the perspective of integrity.

The concept of integrity has various meanings, but in the context of the right to be protected, integrity refers to the physical wholeness of being as it is, rather than the moral virtues such as honesty and purity. In this sense, the fact that X has RBI means that, unless there are other conditions to be considered, others have an obligation not to invade or infringe on X's body. In recent discussions, RBI is understood as “a right that protects against nonconsensual bodily interferences.”^26^ The moral right to be protected from bodily trespass seems to be very fundamental to human beings. Thomson explains this intuition by stating that for a human being, one's body is the most fundamental property, in that it is primary property, and if this is not properly protected, the value of other rights that someone possesses virtually disappears.^27^ The general acceptance of the norm prohibiting torture also demonstrates that respect for RBI is deeply embedded in today's ethical intuition.

It can be reasonably argued that BME involves intervention on the body. Consequently, the coercion of BME for psychopathic offenders will inevitably lead to a violation of RBI. Although there are varying degrees of intervention, invasive brain surgery and even administering drugs by injection or taking pills, if done coercively, will cause a significant level of physical intervention. Those in favor of coercive BME may counter that BME is not analogous to corporal punishment or torture, which causes pain or damage to the body. Rather, they argue, it is an attempt to promote bodily integrity by improving bodily dysfunctions. However, as previously stated, in this context, integrity means the wholeness of being as it is. Consequently, whether bodily functions are diminished or enhanced, if an invasion against one's will occurs, RBI can be violated. If an act of intervening in the body can be justified by the intention or result of improving bodily functions, it would also justify various forced treatments that are considered unethical. In such cases, the relevant right would become a nominal one that fails to protect the individual's interests.

It is important to note that, like other rights, the right to bodily integrity (RBI) is not an absolute right. In the context of this study, there is a special consideration of intervention for psychopathic offenders, for whom there is limited potential for improvement by other means.^28^ Therefore, it is difficult to conclude that BME for psychopathic offenders is completely impermissible simply because it involves non‐voluntary bodily invasion, especially if the intervention does not clearly cause significant damage or harm, at least from the perspective of the physical body. To substantiate the argument that BME coercion is impermissible, it is necessary to demonstrate that it causes unjustifiable negative consequences that are distinct from the violation of bodily integrity. The violation of mental integrity and the right to it is precisely that.

The concept of mental integrity can be derived in two ways. The first is an analytical and logical way, which is to understand mental integrity as one of the two sub‐concepts of personal integrity along with bodily integrity. The second is a holistic and empirical approach, which is to show that the concept of body dealt with in the context of today's integrity discussion is a term that encompasses both the physical and psychological elements of human beings. Mental integrity and the right to mental integrity (RMI) that it entails have historically been protected under the name of “freedom of thought” or “freedom of conscience,” and as neuroscience advances and concerns grow about direct intervention, violation, and manipulation of the human mind, its importance is being further emphasized.^29^ Regardless of the judgment on which of the above two ways is more appropriate to derive the concept of “mental integrity,” RMI seems to be a concept that can be sufficiently justified in today's normative horizon.

Let us assume there is a psychopathic offender P1 who refuses BME. P1 is a typical psychopathic offender, exhibiting an array of abnormalities in emotional functions that regulate antisocial criminal behavior. Given his average level of instrumental rationality, he is able to comprehend his own dysfunctions and the fact that, as a consequence, he encounters difficulty in inhibiting his behavior. Consequently, he is likely to persist in committing crimes in the future and, as a result, to remain in a state of constant conflict with, and isolation from, society. Furthermore, he is aware that meaningful improvement can be achieved through BME and that the improvement effect of BME can positively influence the judgment of his recidivism rate and the parole decision based on it. Nevertheless, P1 refuses BME. In this case, P1 can be evaluated as having a strong belief that the wholeness of his current antisocial self should be maintained or that his antisociality should not be improved by state coercion. Since P1 adheres to this belief despite predictable practical benefits such as early release from imprisonment, this belief seems to be a core aspect that constitutes, at least in that context, his mental integrity. If the state forces BME on such P1, his RMI will be violated, in that the belief that forms the core of mental integrity is seriously damaged.

Of course, RMI, like other rights, can be limited for reasons such as conflicts with other rights. However, when a right is limited, the severity and extent of the restriction and the suitability of the means, which are closely related to it, need to be carefully considered. The most immediate and direct goal of coercive BME for psychopathic offenders is to prevent future harm and rights violations. While BME may be a promising means for this, coercive BME will result in serious harm to mental integrity, as discussed thus far. On the other hand, the goal of preventing harm and rights violations can be achieved to some extent by the institutional means traditionally employed to separate psychopathic offenders from society, such as imprisonment or confinement. Therefore, for psychopathic offenders who clearly refuse BME, the use of these traditional penal systems would be more appropriate. This perspective is divergent from the assertion that RMI is objectively more significant than RBI. My point is that, in a situation where one of two rights someone has must be restricted and both rights are important, it is necessary to respect the opinion of the party whose rights are being restricted as much as possible.

## 5 SCENARIO 3: VOLUNTARY BME FOR PSYCHOPATHIC OFFENDERS

As mentioned earlier, there is the position against even voluntary BME in the case of psychopathic offenders.^30^ Hübner and White argue that performing neurosurgical procedures such as deep brain stimulation (DBS) on psychopathic prisoners cannot meet “two essential bioethical criteria, individual medical benefit and voluntary informed consent.”^31^ Similar to the position of this study, they also view, based on several studies, that what psychopaths generally lack is not the theoretical ability of the cognitive aspect of morality, but the motivation to follow morality. They acknowledge the potential efficacy of technologies such as DBS in improving or alleviating psychopathic traits. However, they argue that psychopaths are completely satisfied with their own identity and thus lack internal motivation to improve their psychopathic disposition. In other words, psychopaths are content with their psychopathic tendencies and do not exhibit an intrinsic desire to overcome this condition. Consequently, Hübner and White argue that DBS offers no tangible medical benefits to the individual. In such a context, if incarcerated psychopathic offenders were to consent to a risky neurosurgical procedure like DBS, it would be for extrinsic benefits such as reduced punishment, rather than for the amelioration of psychopathy per se. From the authors’ perspective, these benefits represent undue influence that poses a significant risk to the respect for the autonomy of vulnerable groups. Even if the intervention is carried out with explicit consent, it cannot be recognized as voluntary from a bioethical perspective.

Before refuting the above argument, it is necessary to recall my assumption, safe and effective BME, once again. In this respect, Hübner and White's position may be seen as significantly undermining their argument if the development of safe technology is assumed, as they strongly emphasize the inherent risk and side effects of DBS. However, their argument is not based solely on the risk of DBS. From an ethical point of view, their more core premises are as follows:

(1) Psychopaths are completely satisfied with their identity.
(2) Thus, neural interventions for psychopathic prisoners do not provide individual medical benefits.
(3) In the absence of individual medical benefits, the voluntariness of BME consent cannot be recognized.

These premises are grounds that are independent of the risk of DBS. Therefore, they must be examined independently, even from my position that assumes the safety of biomedical interventions. Logically, (2) and (3) depend on (1). Therefore, if (1) is denied, (2) and (3) are also denied in turn. In fact, recent research related to the treatment of psychopathic offenders argues that psychopaths can be committed to programs that help them better control and manage antisocial behavior. This strategy is actually effective, and in this approach, an individualized approach and the formation of a therapist–patient alliance plays an important role.^32^ From an ethical standpoint, nevertheless, it is more intriguing to demonstrate that (2) and (3) or their implications can be dismissed while accepting (1). Consequently, the subsequent discussion will proceed in a manner that accepts (1) but rejects (2) and (3) or their implications.

Two implicit propositions are posited between (1) and (2). The first (a) is that psychopathy is not associated with subjective suffering. The second (b) is that “individual medical benefit requires release from, or prevention of, subjective suffering on the side of the person concerned.”^33^ For the purposes of this discussion, we will accept the first proposition, which is considered an implication of (1) that we have decided to accept. We will then critically examine the second proposition. The authors acknowledge that there are scholars who challenge the classification of psychopathy as a disease or disorder. However, they propose that psychopathy exhibits pathological characteristics according to strict criteria, which include an objective diagnosis and treatment as a disease or disorder. Furthermore, they suggest that effective treatment is also possible.^34^ Nevertheless, by assuming that the liberation from or prevention of subjective distress is a necessary condition for recognizing medical benefits, they argue that psychopathy is a disease, but that treating it does not provide individual medical benefits.

This perspective appears to lack consistency. The approach to consider psychopathy as a disorder or disease, despite the absence of distress or discomfort in the individual, implies an attempt to understand disease as an objective concept, not a subjective one. Boorse defined disease as “internal states that depress a functional ability below species‐typical levels” from the perspective of biological function and statistical normality and argued that this concept is value‐free.^35^ If such an objective view is taken on the diagnosis and treatment effects of disease, it seems more coherent to take an objective view on the benefits of medicine as well. For example, if compulsory education is objectively beneficial, in that it facilitates the acquisition of basic knowledge, even if the individual concerned does not have any internal desire related to what they have acquired, it can be evaluated that they have benefited from receiving such education. If an objective perspective is taken on disease and treatment, a subjective view on medical benefits is not coherent. If psychopathy is a disease that can be objectively diagnosed and treated, regardless of the subjective judgment of the individual concerned, it can be sufficiently evaluated as bringing medical benefits to that individual.

Of course, coherence can be a matter of degree, as there is no logical contradiction in their argument. Moreover, the authors’ concern about social considerations, such as recidivism prevention, overwhelming the individual deserves to be sufficiently emphasized. Therefore, let us assume that (1) and (2), as well as (a) and (b), are all valid and examine (3). What (3) implies is that individual medical benefit is a necessary condition for recognizing the voluntariness of consent and providing treatment‐external incentives such as reduction or exemption from punishment without this condition being secured is, instead, an undue influence that threatens their self‐determination as a vulnerable group. It is ethically challenging to permit proposing biomedical interventions to those in vulnerable positions for social benefits alone without individual benefits. However, the limitation of individual benefits to medical benefits is problematic.

In the case of a general clinical situation regarding the issue of poverty and vulnerable individuals, the argument that only medical benefits or intra‐medical benefits should be used as a criterion for judging the voluntariness of consent would be more persuasive. However, the choice situation of a psychopathic offender regarding BME should be distinguished from general situations such as human experiments on vulnerable individuals due to poverty. Unlike the latter, the former is also in a criminal context, in that it is the state's proposal to the offender. In this overlapping context, a psychopathic prisoner's decision is not solely a matter of medical decision‐making, but also an issue of a rational individual within the criminal justice system utilizing their prudent abilities fully to select what is best for themselves. If the inmate's choice of BME results in both medical benefits and advantages within the criminal context, it would be ideal, and this case certainly allows for such an interpretation. However, even if we assume, as the authors suggest, that only the latter is provided without the former, if the inmate themselves clearly recognizes this situation and rationally considers it before consenting to BME, it would be appropriate for us to respect such a judgment. If one were to argue that the medical context is more fundamental between the two, and thus that medical benefits are a necessary condition for recognizing the voluntariness of consent to BME, this would appear to be a manifestation of difficult‐to‐justify moralistic paternalism. If the true aim is to respect the autonomy of incarcerated psychopaths through securing “voluntary informed consent”, then we should acknowledge and permit their exercise of agency in ascribing high value to criminal justice benefits.

It is evident that the concern regarding the external benefits of treatment is not completely wrong. For BME consent to be truly voluntary, there must be scope to refuse BME, and excessively strong incentives will not leave such scope.^36^ Therefore, it would be better for the BME proposal to be made after the sentence has been determined. Moreover, promising parole in exchange for the implementation of BME itself also seems to be problematic. This may be giving privileges not offered to other inmates. This is because existing parole decisions are made based on criteria such as reduced risk of recidivism. In our discussion, BME will be effective in improving psychopathy, but the extent of effectiveness may vary. There is a big difference in the actual meaning and influence on decision‐making between suggesting to an inmate that “parole will be decided by a comprehensive assessment of improvement effect and risk of recidivism after BME” and suggesting “you will be paroled X months after BME.” As mentioned above, the latter can be considered an excessive incentive because it is a privilege not offered to other inmates.

In conclusion, the most ethically desirable approach that aligns with existing criminal justice systems is to refrain from offering independent incentives directly tied to the consent for (or actual implementation of) BME. In this context, the benefit that a psychopathic offender might rationally consider in relation to BME consent is the increased likelihood of parole. Of course, it should be evaluated through the same procedures currently employed within the existing criminal justice system. This can indeed be regarded as a prudent benefit, as it would be difficult to attain without consenting to BME. Moreover, this approach utilizes the same parole system and assessment criteria that the existing criminal justice system already applies to other general prisoners. Consequently, it is relatively immune to criticism of being excessively preferential, as it does not provide special treatment for BME. Under these conditions, voluntary BME for psychopathic offenders appears to be ethically permissible.

## 6 CONCLUSION

In this article, I have examined the ethical permissibility of coercive and voluntary BME for psychopaths. I first explained the concept of psychopathy and its normative implications. Based on that, I analyzed three scenarios of BME for psychopaths. The objective was to demonstrate that the existing intuition that while coercive BME is ethically impermissible, voluntary BME can be ethically permissible can be held even in the case of psychopaths.

The most crucial factor for the practical application of BME is the development of safe technology. The scope of voluntary BME will require further discussion in specific cases. For example, would nudging psychopaths to increase consent be considered in the context of voluntary BME? Additionally, it would be beneficial to consider changes in the general public's view of criminal justice and legal sentiments. It is important to note that all of these scenarios are based on a view of justice that is centered on rehabilitation and prevention. On the one hand, this makes it difficult to hold individuals fully responsible for their psychopathic traits and argues that society should help psychopathic offenders quickly and stably reintegrate into society. In countries where retributivism is prevalent, such as those that maintain the death penalty, this approach may not be accepted at all by the public. However, on the other hand, this therapeutic approach might increase the likelihood of psychopaths who refuse BME becoming subject to prolonged or nearly permanent isolation through hospital orders or special verdicts, on the grounds that they have not demonstrated correction. In actual application, such a comprehensive context needs to be considered. It is my hope that this article will contribute to more practical discussions in the future.

## Footnotes

1 This functional definition can include many kinds of interventions, from drug injections to neurosurgery. In this definition, the concept of enhancement means the broad sense of improvement in general, which includes the concept of treatment.

2 Persson, I., & Julian S. (2008). The perils of cognitive enhancement and the urgent imperative to enhance the moral character of humanity. *Journal of Applied Philosophy*, *25*(3), 162–177; Persson, I., and Julian S. (2012). *Unfit for the Future: The Need for Moral Enhancement*. Oxford University Press.

3 Harris, J. (2011). Moral Enhancement and Freedom. *Bioethics*, *25*(2), 102–111; Hauskeller, M. (2017). Is it desirable to be able to do the undesirable?: Moral bioenhancement and the little Alex problem. *Cambridge Quarterly of Healthcare Ethics*, *26*(3), 365–376.

4 Vedder, A. (2019). An obligation to enhance? *Topoi*, *38*, 49–52; Kudlek, K., & Patrick T. S., (2022). The Kantian promise and peril of moral bioenhancement. *Journal of Applied Philosophy*, *39*(3), 487–503.

5 Schaefer, G. O. (2015). Direct vs. indirect moral enhancement. *Kennedy Institute of Ethics Journal*, *25*(3), 261–289; Rakić, V. (2014). Voluntary moral enhancement and the survival‐at‐any‐cost bias. *Journal of Medical Ethics*, *40*(4), 246–250.

6 Baccarini, E., & Malatesti, L. (2017). The moral bioenhancement of psychopaths. *Journal of Medical Ethics*, *43*(10), 697–701.

7 Hübner, D., & Lucie W. (2016). Neurosurgery for psychopaths? An ethical analysis. *AJOB Neuroscience*, *7*(3), 140–149.

8 If we distinguish between offenders and non‐criminals in terms of BME subjects, and voluntary and coercive in terms of BME consent, four possible cases arise in a 2 × 2 matrix. However, in this paper, the scenario of “voluntary BME for non‐criminal psychopaths” is excluded. This is due to my view that it does not need to be dealt with separately from the existing discussions on the ethical permissibility of voluntary BME.

9 Hare, R. D. (1999). *Without conscience: The disturbing world of the psychopaths among us* (p. 25). Guilford Press.

10 Baron‐Cohen, S. (2012). *Zero degree of empathy: A new theory of human cruelty* (p. 46). Penguin Books.

11 Blair, J., Mitchell, D., & Blair, K. (2005). The psychopath: Emotion and the brain (p. 6). Blackwell Publishing; Hare, op. cit. note 9, p. 24.

12 ibid.

13 Baron‐Cohen, op. cit. note 10, p. 61.

14 Carter, S. (2017). Could moral enhancement interventions be medically indicated? *Health Care Analysis*, *25*, 345.

15 Fallon, J. (2014). *The psychopath inside: A neuroscientist's personal journey into the dark side of the brain* (p. 160). Penguin.

16 ibid.

17 The most recent systematic contribution to the discourse on moral responsibility in psychopathy has been made by Jim Baxter (2021). Baxter extends the discussions of Strawson and Wallace to propose “responsiveness to reasons” as a prerequisite for moral responsibility. Drawing on multidisciplinary research, he presents a compelling argument that deficits in emotional functioning and development in psychopaths impair their ability to respond to the rationale “not to harm others.” Specifically, Baxter contends that these impairments prevent psychopaths from internally recognizing this rationale and modulating their behavior accordingly. See Baxter, J. (2021). *Moral responsibility and the psychopath: The value of others*. Cambridge University Press.

18 Recent neurobiological research on psychopathy has identified promising therapeutic interventions, including pharmacological approaches to balance cortisol and testosterone levels and the use of repetitive transcranial magnetic stimulation (rTMS) to improve orbitofrontal cortex function and reduce impulsivity; Glenn, A., and Adrian R., (2008). “The neurobiology of psychopathy.” *Psychiatric clinics of North America*, *31*, 470–471.

19 Thomson, J. J. (1975). The right to privacy. *Philosophy & Public Affairs*, *4*(4), 295–314.

20 Altman, I. (1976). A conceptual analysis. *Environment and Behavior*, *8*(1), 7–29; Steeves, V. (2009). Reclaiming the social value of privacy. In I. Kerr, V. Steeves, & C. Lucock (Eds.), *Lessons from the identity trail: Anonymity, privacy and identity in a networked society* (pp. 191–208). Oxford University Press.

21 For a comprehensive conceptualization of stigma, see Link, B. G., & Jo, C. P. (2001). Conceptualizing stigma. *Annual Review of Sociology*, *27*(1), 363–385.

22 Corrigan, P. W., Amy C. W., & Leah B. (2006). The self–stigma of mental illness: Implications for self–esteem and self–efficacy. *Journal of Social and Clinical Psychology*, *25*(8), 876.

23 Moore, K. E., Katherine C. M., Johanna B. F., and June P. T. (2018). Self‐stigma among criminal offenders: Risk and protective factors. *Stigma and Health*, *3*(3), 241. It seems a reasonable speculation that prosocial psychopaths could, as an unintended consequence of this process, develop antisocial behaviors. However, ethical considerations would preclude the possibility of conducting an actual human experiment to test this hypothesis.

24 Dennett, D. C., & Gregg, D. C. (2021). *Just deserts: Debating free will* (p. 141). Polity Press.

25 In this regard, Douglas argues that the coercion of medical interventions or corrections might be permissible under certain circumstances, based on the fact that non‐consensual incarceration of offenders is widely justified. Douglas, T. (2014). Criminal rehabilitation through medical intervention: Moral liability and the right to bodily integrity. *The Journal of Ethics*, *18*, 101–122.

26 Tesink, V., Thomas D., Lisa F., Sjors L., and Gerben M. (2023). Neurointerventions in criminal justice: On the scope of the moral right to bodily integrity. *Neuroethics*, *16*(3), 4.

27 Thomson, J. J. (1990). *The realm of rights*. (pp. 205–226). Harvard University Press.

28 Current treatment programs for violent offenders, which typically emphasize anger control and social skills, have demonstrated reduced effectiveness when applied to psychopaths. Freberg, L. A. (2018). *Discovering behavioral neuroscience: An introduction to biological psychology* (4th ed.). (p. 589). Cengage.

29 Lavazza, A., & Rodolfo G. (2023). Philosophical foundation of the right to mental integrity in the age of neurotechnologies. *Neuroethics*, *16*, 10.

30 Hübner & Lucie, op. cit. note 7.

31 Ibid: 140.

32 de Ruiter, C., & Martin H. (2022). Therapeutic Considerations and Interventions for Psychopathy. In J. E. Vitale (Ed.), *The complexity of psychopathy* (pp. 359–380). Springer International Publishing.

33 Hübner & Lucie, op. cit. note 7, pp. 141–143.

34 Ibid: 143.

35 Boorse, C. (1977). Health as a theoretical concept. *Philosophy of Science*, *44*(4), 542.

36 For the problem of undue inducement, see Beauchamp, T. L., & James F. C. (2012). *Principles of biomedical ethics* (7th ed.) (pp. 268–269). Oxford University Press.

